# MoWa: A Disinfectant for Hospital Surfaces Contaminated With Methicillin-Resistant *Staphylococcus aureus* (MRSA) and Other Nosocomial Pathogens

**DOI:** 10.3389/fcimb.2021.676638

**Published:** 2021-07-06

**Authors:** Tyler V. Gregory, Karen Ellis, Renzo Valeriani, Faidad Khan, Xueqing Wu, Landon Murin, Babek Alibayov, Ana G. Jop Vidal, Tong Zhao, Jorge E. Vidal

**Affiliations:** ^1^ Department of Microbiology and Immunology, University of Mississippi Medical Center, Jackson, MS, United States; ^2^ Biomedical Sciences Master of Science Program, University of Mississippi Medical Center, Jackson, MS, United States; ^3^ Rollins School of Public Health, Emory University, Atlanta, GA, United States; ^4^ Department of Infectious Disease, Sir Run Run Shaw Hospital, College of Medicine, Zhejiang University, Hangzhou, China; ^5^ Base Pair Program Murrah- University of Mississippi Medical Center, Jackson, MS, United States; ^6^ Center for Food Safety, University of Georgia, Griffin, GA, United States

**Keywords:** methicillin-resistant *Staphylococcus aureus*, disinfectant, *Staphylococcus aureus*, nosocomial pathogens, contaminated surface

## Abstract

**Introduction:**

*Staphylococcus aureus* strains, including methicillin-resistant *S. aureus* (MRSA) and methicillin-sensitive *S. aureus* (MSSA), are a main cause of nosocomial infection in the world. The majority of nosocomial *S. aureus*-infection are traced back to a source of contaminated surfaces including surgery tables. We assessed the efficacy of a mixture of levulinic acid (LA) and sodium dodecyl sulfate (SDS), hereafter called MoWa, to eradicate nosocomial pathogens from contaminated surfaces.

**Methods and Results:**

A dose response study demonstrated that MoWa killed 24 h planktonic cultures of *S. aureus* strains starting at a concentration of (LA) 8.2/(SDS) 0.3 mM while 24 h preformed biofilms were eradicated with 32/1.3 mM. A time course study further showed that attached MRSA bacteria were eradicated within 4 h of incubation with 65/2 mM MoWa. Staphylococci were killed as confirmed by bacterial counts, and fluorescence micrographs that were stained with the live/dead bacterial assay. We then simulated contamination of hospital surfaces by inoculating bacteria on a surface prone to contamination. Once dried, contaminated surfaces were sprayed with MoWa or mock-treated, and treated contaminated surfaces were swabbed and bacteria counted. While bacteria in the mock-treated samples grew at a density of ~10^4^ cfu/cm^2^, those treated for ~1 min with MoWa (1.0/0.04 M) had been eradicated below limit of detection. A similar eradication efficacy was obtained when surfaces were contaminated with other nosocomial pathogens, such as *Klebsiella pneumoniae*, *Pseudomonas aeruginosa*, *Acinetobacter baumannii*, or *Staphylococcus epidermidis*.

**Conclusions:**

MoWa kills planktonic and biofilms made by MRSA and MSSA strains and showed great efficacy to disinfect MRSA-, and MSSA-contaminated, surfaces and surfaces contaminated with other important nosocomial pathogens.

## Introduction

During the last decade, *Staphylococcus aureus*, including methicillin resistant (MRSA) strains, have remained the main cause of hospital-acquired infection (HAI), i.e., nosocomial, in the United States and throughout the world (Magill et al., 2014; Magill et al., 2018). *S. aureus* strains persist in hospital environments for up to 7 months with no differences found whether the strain bear resistance to antibiotics or not (Kramer et al., 2006; Dancer, 2014). In hospitals where MRSA strains are endemic, bacteria have been isolated from 40% of overbed tables, 27% from beds or siderails, and >20% from door handles (Dancer, 2008). This high prevalence of contamination has placed *S. aureus* strains as the most prevalent etiology in bloodstream infection in the USA (Diekema et al., 2019), and a very common cause of HAI in burn patients (Pangli and Papp, 2019). Outbreaks of MRSA infection have been also reported in neonatal intensive care units (Kim et al., 2019). Along the same lines, other Gram-positive and Gram-negative bacteria that cause a high burden of hospital acquired infections include *Klebsiella pneumoniae*, *Staphylococcus epidermidis*, and *Acinetobacter baumannii* (Magill et al., 2014; Magill et al., 2018; Cruz-Lopez et al., 2020).

It is widely recognized that the environment is a vector that facilitates the transmission of nosocomial pathogens. The environment become contaminated with bacteria shed by hospital staff, patients, and their relatives, and persist in the environment for months (Kristinsdottir et al., 2019; Cruz-Lopez et al., 2020). Hospital staff and visitors are prone to touch these surfaces and, if contaminated, increase the risk of self-infection and transmission of pathogens to others (Facciola et al., 2019). A high touch surface contaminated with *S. aureus* will transmit strains to all (100%) individuals that become in physical contact with the surface (Kramer et al., 2006; Lei et al., 2020).

Current formulation to decontaminate hospital environments utilize sodium hypochlorite (bleach)-derivative solutions (Rutala and Weber, 1997). While these solutions are effective on reducing the bacterial density of contaminants, they are in the long run toxic for the hospital personal due to their oxidizing capacity and the pH of the solution (Slaughter et al., 2019). Moreover, shortages of disinfectants and sanitizers had occurred during the Covid-19 pandemic (Berardi et al., 2020). Because of the abovementioned and the potential toxicity of traditional cleaning methods to decontaminate hospital surfaces, new approaches are needed to assist in reducing the burden of hospital-acquired infections.

A solution made with levulinic acid [1 M (LA)] and sodium dodecyl sulfate [0.5 M (SDS, also known as lauryl sulfate)] has proven effective to sanitize produce contaminated with *Salmonella* spp., *Listeria* spp., and enterohemorrhagic *Escherichia coli* (EHEC), and to inactivate influenza A virus and surrogates for human norovirus (Zhao et al., 2009; Cannon et al., 2012; Aydin et al., 2013; Zhou et al., 2020). This LA/SDS (1 ;M/0.04 M) solution reduced the density of *Salmonella* species by >7 logs and the density of shiga toxin-producing *E. coli* strains by >5 logs within minutes (Zhao et al., 2009; Zhao et al., 2010; Zhao et al., 2014). The efficacy was similarly observed in pure cultures, or when strains were spiked on different surfaces or food items (Zhao et al., 2011; Chen et al., 2014). A similar formulation of LA- and SDS-disinfected bacteria from produce such as pecans (Beuchat et al., 2012; Beuchat et al., 2013), cantaloupes (Webb et al., 2013), or those contaminating beef cheek meat (Schmidt et al., 2014) and eradicated bacteria from food slicers (Chen et al., 2014). However, the same LA/SDS solution was ineffective to eradicate parasites, such as *Cryptosporidium parvum* and *Encephalitozoon intestinalis* (Ortega et al., 2011). To the best of our knowledge, this LA/SDS solution has not been assessed to disinfect clinical settings contaminated with nosocomial pathogens. It has, however, demonstrated potential to remove biofilms made by oral streptococci as assessed by using an *in vitro* approach and a mouse model of oral colonization (Wang et al., 2012).

LA is synthesized by heating hexoses (glucose, fructose) or starch in a diluted acid solution; the final compound is also known as laevulose (Ren et al., 2015; Shen et al., 2015). LA is therefore relatively nontoxic, with an LD50 of 1850 mg/kg for rats and 5000 mg/kg for rabbits (Anonymous, 1979). SDS is a detergent organosulfate widely used for cleaning applications. Both LA and SDS are generally recognized as safe (GRAS) ingredients for food use according to the USFDA (21 CFR 172.822 and 172.515) (Administration FFaD, 2021). Furthermore, these two compounds are approved as food additives, including as a flavoring substance in the case of LA, or as a multipurpose additive for SDS (Zhou et al., 2019; Administration FFaD, 2021).

In this study, we thoroughly characterized the use of the LA/SDS solution as a disinfectant against *S. aureus* strains and other nosocomial pathogens using and *in vitro* approach and also using a more realistic infection model that recreated the contamination of hospital surfaces with nosocomial pathogens. The low cost of both LA and SDS and their availability from multiple reputed vendors are additional advantages for their use in hospitals. Because this solution containing levulinic acid and SDS proved effective for MRSA remOval by simply spraying it (i.e., WAshing) on contaminated surfaces, for the sake of simplicity we have called it “MoWa” throughout this manuscript.

## Materials And Methods

### Strains, Culture Media, and Reagents

All bacterial strains, including MRSA strains, utilized in the study are listed in **Table 1**. Bacteria were routinely cultured in brain heart infusion (BHI) broth or BHI agar plates. Sodium dodecyl sulfate (SDS) and levulinic acid (LA) were sourced from Sigma-Aldrich and solubilized using milliQ water pH = 6.998.

**Table 1:** Bacterial strains utilized in this study.

Strain	Characteristics	Known resistance to antibiotics*	Reference or source
NRS384	USA300, MRSA (*mecA* ^+^);−Strain associated with outbreaks from correctional facilities in MS, GA, TN, TX, and CA	PEN, ERY, OXA	(McDougal et al., 2003)
NRS230	MSSA (*mecA* ^-^), associated with elbow arthritis with scarlet fever	PEN, ERY	Laboratory collection
NRS236	MSSA (*mecA* ^-^), associated with bacteremia	PEN, ERY	Laboratory collection
NRS242	MSSA (*mecA* ^-^), Associated with impetigo	PEN, ERY	(Ray et al., 2016)
NRS408	MRSA (*mecA* ^+^)	PEN, CLI, ERY, GEN, OXA, TMP, TEC, TET	(Flannagan et al., 2003) (Weigel et al., 2003)
NRS49	MRSA (*mecA* ^+^), associated with bacteremia.	PEN, OXA, TET, CIP	(Kim et al., 2011)
*K. pneumoniae* 4/484	Strain isolated from hospital acquired infection (ventilator associated pneumonia)	SAM, FEP, CRO, CAZ, CIP, GEN, TOB, SXT	(Ostria-Hernandez et al., 2018)
*K. pneumoniae* RGV2	Strain isolated from hospital acquired infection (bacteremia)	AMP	(Ostria-Hernandez et al., 2018)
*P. aeruginosa* ATCC BAA-47 (PAO1)	Reference strain, isolated from an infected wound with moderate virulence	AMP	(Lee et al., 2006)
*P. aeruginosa* PA14	Reference strain, isolated from an infected wound with high virulence	AMP	(He et al., 2004)
*A. baumannii* ATCC17978	Genome sequenced strains, isolated from a case of meningitis of a 4-month-old infant	Unknown	(Smith et al., 2007)
*S. epidermidis* ATCC14990	Type strain isolated from the nose.	None	Laboratory collection

*AMP, ampicillin; SAM, ampicillin-sulbactam; FEP, cefepime, CRO, ceftriaxone; CAZ, ceftazidime; CIP, ciprofloxacin; CLI, clindamycin; ERY, erythromycin; GEN, gentamicin; OXA, oxacillin; PEN, penicillin; TEC, teicoplanin; TET, tetracycline;, TOB, tobramycin; TMP, trimethoprim; SXT, trimethoprim-sulfamethoxazole.

### Preparation of Inoculum for Experiments

Inoculum was prepared essentially as previously described (Wu et al., 2017; Wu et al., 2019). Briefly, an overnight culture on BHI agar plate was used to prepare a cell suspension in BHI broth to an OD_600_ of ~0.08. This suspension was incubated at 37°C in a 5% CO_2_ atmosphere until the culture reached an OD_600_ of ~0.2 (early-log phase). The culture was added with glycerol to give a final 10% (v/v) concentration and stored at −80°C until used. The day before the experiments, an aliquot of these bacterial stocks was removed from the freezer and diluted and plated to obtain bacterial counts (cfu/ml). A similar inoculum has been utilized in studies of other human pathogens (Vidal et al., 2011; Vidal et al., 2013; Wu et al., 2017; Ostria-Hernandez et al., 2018).

### Treatment of MoWa to Planktonic and Biofilms

All experiments in these sections were inoculated with *S. aureus* MSSA and MRSA strains at a density of ~1 × 10^6^ cfu/ml, using either a six-well plate or 24-well plate containing BHI as indicated in each experiment. Experiments were repeated at least three times. As per definition, the MIC_90_ was the concentration of MoWa at which ≥90% of isolates tested were killed, under the challenge conditions assessed in this study (Schwarz et al., 2010).

i) Planktonic cells challenged with MoWa. Bacteria were inoculated and treated with different dosages of MoWa, of left untreated, and incubated for 24 or 1 h at 37°C in a 5% CO_2_ atmosphere. Planktonic bacteria were then diluted and plated onto BHI agar plates to obtain bacterial counts (cfu/ml).

ii) Biofilms and attached MRSA challenged with MoWa. Staphylococci were inoculated as mentioned above and incubated for 24 h to form mature biofilms, or for 4 h to allow MRSA bacteria attach the substratum. Planktonic bacteria were then removed, and biofilms that were attached to the substratum, were gentle washed two times with sterile PBS and added with fresh BHI. Biofilms were then treated with different dosages of MoWa, or left untreated, for the indicated time (i.e., 24, 12, 4, or 1 h) at 37°C in a 5% CO_2_ atmosphere. At the end of the incubation, 24 h biofilms or 4 h attached bacteria were washed twice with sterile PBS, resuspended in 1 ml of sterile PBS, and sonicated for 15 s using a Bransonic ultrasonic water bath (Branson, Danbury, CT), followed by extensive pipetting to remove remaining attached biofilm bacteria. Biofilms were then diluted and plated onto BHI agar plates to obtain bacterial counts (cfu/ml).

### Evaluation of MRSA Viability Using the Live/Dead Cell Assay

An aliquot (~1 × 10^6^ cfu/ml) of the MRSA strain was inoculated in duplicate into either an eight-well glass slide (Lab-Tek, Rochester, NY) containing BHI broth and incubated at 37°C with 5% CO_2_ for 4 or 24 h. Planktonic bacteria were removed as above, and 4 h attached MRSA bacteria, or 24 h biofilms, were treated with the indicated dose of MoWa, or left untreated (control), and incubated for 4 or 24 h, at 37°C in a 5% CO_2_. A LIVE/DEAD BacLight bacterial viability kit L7012 (Invitrogen-Molecular Probes) was used to visualize the viability of MRSA biofilms. Fluorescent dyes included in the kit incorporate, or not, into bacterial cells as a function of membrane integrity, and therefore viability. Staining procedure and concentration of dyes were utilized as per manufacturer’s recommendations. Preparations with the 4-h experiments were observed and photographed utilizing an upright, epi-fluorescence, Nikon Ni-U Research Microscope equipped with a Nikon DS-Qi2 sCMOS Camera system. Preparations with the 24-h experiments were imaged using an Olympus FV1000 confocal microscope and analyzed with ImageJ version 1.49k (National Institutes of Health, USA).

### Contaminated Surface Model

Strains were inoculated at a density of ~1 × 10^6^ in a dedicated 10 sq. cm. area on a laboratory bench. The required volume of the strain was dropped, spread in the surface area using a sterile inoculation loop and allowed to dry for ~1 h. MoWa was applied to each area with a spray bottle which dispensed ~780 µl, as calculated by repeated measurements, containing the MIC_90_. The MoWa-treated contaminated surface was allowed to sit for the indicated time before bacteria were collected using a cotton swab (Fisher Brand) that had pre-wet by introducing the swab into a sterile tube containing 1 ml of BHI broth. The swab was rubbed in a zig-zag pattern throughout the complete area and was rotated three times to assure all bacteria were removed from the surface. The swab was then placed back into the tube with BIH and vortexed for 30 s. This BHI broth was diluted, and dilutions plated on BHI agar plates and incubated at 37°C with 5% CO_2_ for 24 h for colony counts (cfu/ml).

### Statistical Analysis

All statistical analyses were determined using the two-tailed Student *t* test and the software SigmaPlot version 14.0.

## Results

### MoWa Kills Planktonic *S. aureus* strains and Preformed Biofilms

**Figure 1:**
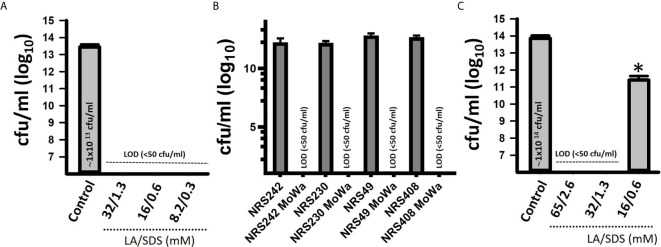
MoWa eradicates planktonic and biofilm cultures of MRSA strains. Bacteria were inoculated (~1 × 10^6^ cfu/ml) in 24-well plates containing BHI broth and left untreated (control) or treated with the indicated dose of a mixture of levulinic acid (LA) and SDS (MoWa). **(A)** Treated and untreated NRSA384 cultures were incubated at 37°C for 24 h and then planktonic staphylococci were harvested, diluted and plated to obtain the density (cfu/ml). **(B)** The indicated MRSA or MSSA strain was left untreated or challenged with MoWa 8.2/0.3 mM. **(C)** Strain NRS384 was inoculated as above and incubated from 24 h. Planktonic staphylococci were then removed, and biofilms were washed and added with BHI broth. These biofilms were treated with the indicated dose of a mixture of levulinic acid (LA) and SDS for 24 h and then biofilms were harvested to obtain colony counts (cfu/ml). Error bars represent the standard errors of the means calculated using data from at least three independent experiments. *p<0.05 compared to control. LOD, limit of detection. In panels **(A, C)** the median density is shown inside the bar of the untreated control.

We first investigated the potential antimicrobial activity of MoWa against *S. aureus* planktonic cells. Cultures of an epidemic clone MRSA USA300 (NRS384), grown to mid-log phase, were treated with different dosages (**Table 2**) of MoWa for 24 h. As shown in **Figure 1**, MoWa at a concentration of levulinic acid and SDS of 8.2 and 0.33 mM, respectively (thereafter referred as 8.2/0.33 mM), was sufficient to kill NRS384 planktonic bacteria 24 h post-inoculation that otherwise grew at a high density of ~1 × 10^13^ cfu/ml (**Figure 1A** and **Table 2**). A similar low concentration of MoWa was enough to eradicate planktonic cultures of other MRSA and methicillin-sensitive (MSSA) strains (**Figure 1B**).

**Table 2:** Antibacterial effect of MoWa (LA+SDS) against MRSA strain NRS38.

Concentration (M)		Incubation time with MoWa to reach a MIC_90_^&^			
MoWa containing a mixture of:		24 h*		4 h	1 h
Levulinic acid (LA)	SDS	Planktonic	Biofilms**	Biofilms	Planktonic/biofilms
2.1	0.085	+	+	+	+
1.05	0.0425	+	+	+	+
0.525	0.02125	+	+	+	−
0.2625	0.010625	+	+	+	−
0.13125	0.005313	+	+	+	−
0.065625	0.002656	+	+	+	−
0.032813	0.001328	+	+	−	−
0.016406	0.000664	+	−	−	−
0.008203	0.000332	+	−	−	−
0.004102	0.000166	–	−	−	−
0.002051	8.3E-05	–	−	−	−

+, eradication of S. aureus MRSA strain NRS384 (US300) below the limit of detection (10 cfu/ml). −, S. aureus MRSA US300 grew at a density corresponding to ≥50% of the density of the untreated control. ^&^All experiments were inoculated with ~1 × 10^6^ cfu/ml. *Experiments were performed in 24-well plates. **Biofilms were performed for 24 h and then challenged with MoWa.

Given the observed high potency of MoWa against planktonic cells, we assessed its antimicrobial activity against MRSA biofilms. A mid-log phase culture of MRSA strain NRS384 was inoculated into a 24-well plate containing fresh BHI broth and incubated for 24 h. After this incubation period, planktonic staphylococci were removed, and biofilms were challenged with the mixture of levulinic acid and SDS at different concentrations (**Table 2**). Treated and untreated biofilms were incubated for another 24 h period. A concentration of levulinic acid and SDS at 32/1.3 mM, respectively, eradicated preformed NRS384 biofilms, whereas the untreated NRS384 biofilm control reached a very high density (**Figure 1C** and **Table 2**).

### Rapid Killing of Attached MRSA Bacteria by Incubation With MoWa

**Figure 2:**
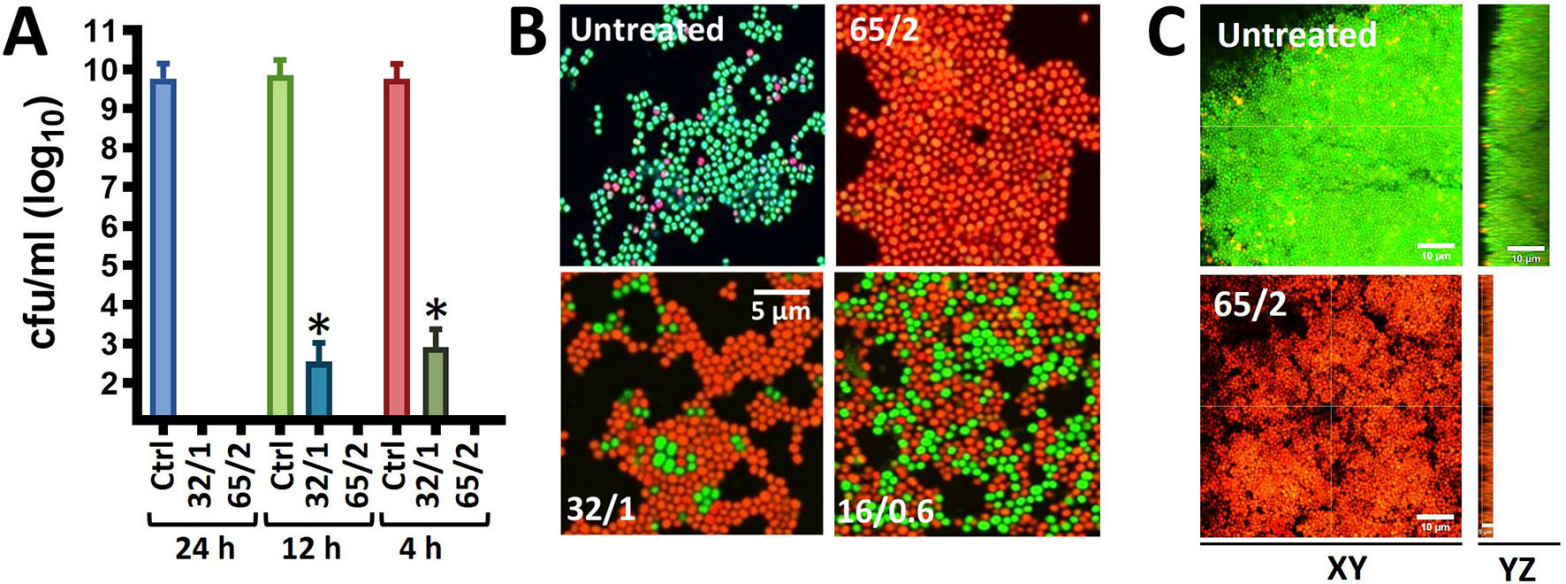
MoWa kills preformed biofilm cultures of MRSA strain NRS384. **(A)** Bacteria were inoculated (1 × 10^6^ cfu/ml) in **(A)** 24-well plates, or **(B)** 8-well slides, containing BHI broth and incubated for 4 h. Planktonic staphylococci were then removed and biofilms were washed and added with BHI broth. These biofilms were left untreated or treated for **(A)** 24 h, 12 h, or 4 h, or **(B)** for 4 h with a mixture levulinic acid (LA) and SDS at a concentration of 65/2 mM, 32/1 mM, or 16/0.6 mM. Biofilms were **(A)** harvested to obtain colony counts (cfu/ml) or **(B)** stained with the LIVE/DEAD BacLight bacterial viability kit and photograph using a Nikon upright fluorescence microscope. Error bars in **(A)** represent the standard errors of the means calculated using data from at least three independent experiments. *p<0.05 compared to control. **(C)** Bacteria were inoculated as above in eight-well slides and incubated for 24 h after which biofilms were treated for 24 h with MoWa 65/2 mM or left untreated. Preparations were stained with the LIVE/DEAD BacLight bacterial viability kit and imaged using a confocal microscope. Panels show XY or YZ optical images.

Given that MoWa can be utilized to remove MSSA and MRSA strains from surfaces contaminated with a lower density to that obtained in 24 h cultures, we further investigated the timing and concentration required to eradicate MRSA bacteria that had attached to the substratum after being cultured for 4 h. Attached MRSA bacteria were treated with different dosages of MoWa, and treated staphylococci were incubated for 24, 12, or 4 h. MoWa at a concentration of 65/2.6 mM eradicated MRSA-attached bacteria as soon as 4 h post-treatment (**Figure 2A** and **Table 2**), whereas a concentration of 32/1.3 mM reduced ~99% the density of MRSA biofilms at the same time point (median, 1.05 × 10^3^ cfu/ml) compared with the untreated control, where the median density was 4.01 × 10^9^ cfu/ml (**Figure 2A**).

To confirm that MoWa had killed 24 h MRSA mature biofilms investigated in experiments presented in **Figure 1**, and 4 h attached bacteria, and not only inhibited their growth on the agar plate, we stained MoWa-treated and untreated MRSA bacteria with the live/dead BacLight bacteria viability assay, and the preparations were observed under a fluorescence microscope. As expected, micrographs of untreated MRSA biofilms showed ≥90% of live staphylococci retaining the cell permeable SYTO-9 green fluorescent reporter, while ≤10% MRSA cells had a damage cell wall and therefore propidium iodide permeated inside those bacteria (**Figure 2B**, upper left panel). MRSA cells treated with a lower dose of MoWa, i.e., 32/1.3, or 16/0.6, showed a mixture of live SYTO-9-positive bacterial cells and dead staphylococci that had incorporated propidium iodide (**Figure 2B**, lower panels). In contrast, MRSA biofilm cells challenged with MoWa, 65/2.6 mM (**Figure 2B**, upper right panel) and higher doses (not shown), were all stained with propidium iodide, indicating that bacteria had been intoxicated and therefore that their membrane was permeabilized (**Figure 2B**). Similarly, whereas 24 h mature MRSA biofilms were viable (**Figure 2C**, XY optical section) and formed a thick ~15 µm biofilm structure (**Figure 2C**, YZ optical section), those treated with MoWa (65/2.6 mM) for 24 h showed a ~75% reduction of thickness (~5 µm) of the biofilm structure and MRSA bacteria that remained attached to the substratum have incorporated propidium iodide confirming that bacteria were dead. Together, these experiments demonstrated that treatment of MRSA bacteria with MoWa 65/2.6 mM for 4 h, or mature biofilms for 24 h, results in dead of staphylococci by a mechanism that involves the permeabilization of the bacterial membrane.

### Minimum Inhibitory Concentration of MoWa Against MRSA and MSSA Strains

**Figure 3:**
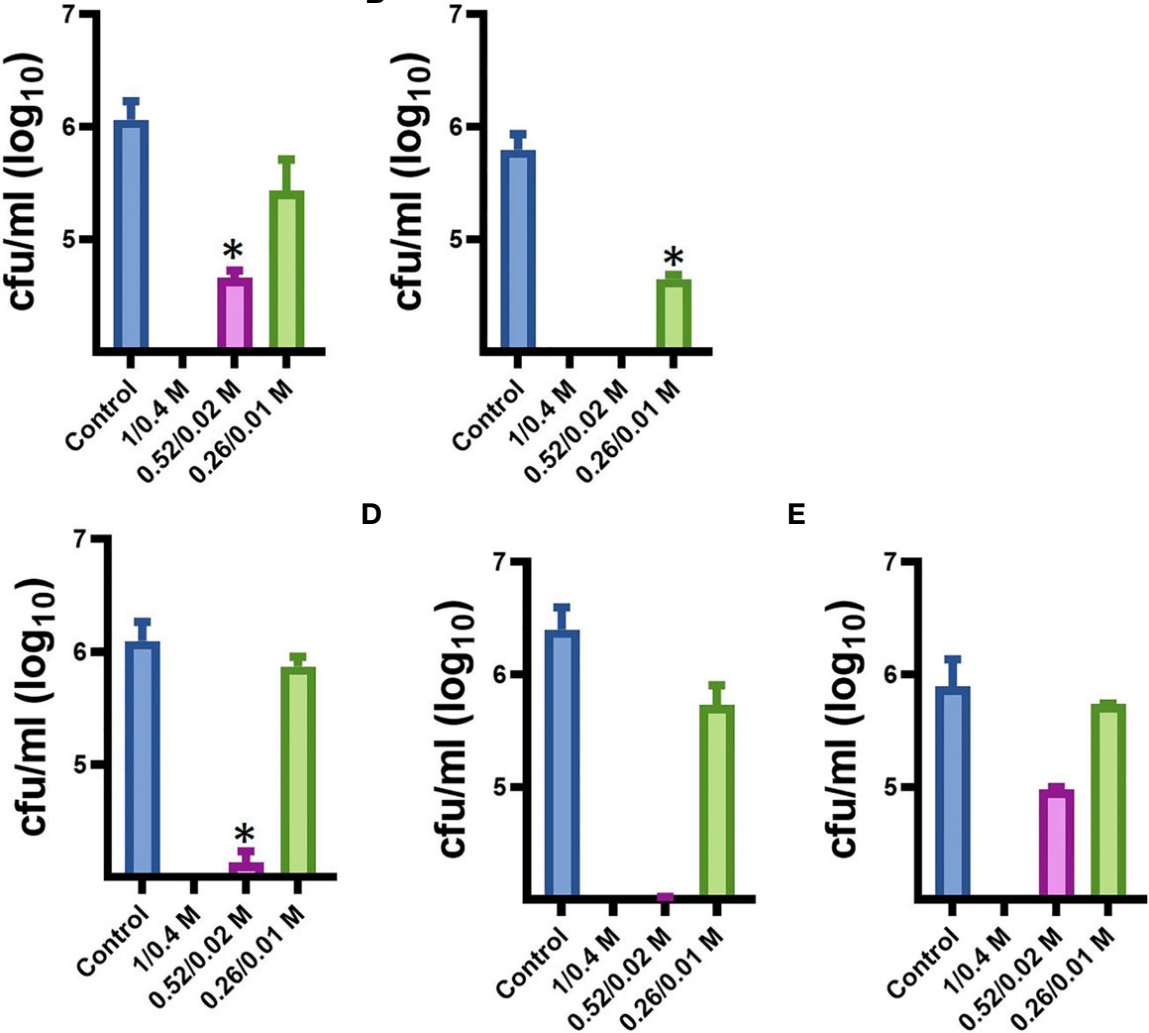
Minimum inhibitory concentration of MoWa against MRSA and MSSA strains. S. aureus strain **(A)** NRS408, **(B)** NRS49, **(C)** NRS236, **(D)** NRS230, or **(E)** NRS242 was inoculated in a six-well plate and treated with a mixture levulinic acid (LA) and SDS at a concentration of 1/0.04 M, 0.52/0.02 M, or 0.26/0.01 M for 1 h. Bacteria were removed, diluted and plated onto BHI agar plates containing 7% of NaCl to obtain colony counts (cfu/ml). Error bars represent the standard errors of the means calculated using data from at least three independent experiments. *p<0.05 compared to control.

Since our next goal was to assess the effectiveness of MoWa as a contact-killing disinfectant, we investigated the MIC_90_ of MoWa that kills *S. aureus* strains within minutes utilizing a collection of MRSA and MSSA strains listed in **Table 1**. Bacteria were inoculated in BHI broth at a concentration of ~1 × 10^6^ cfu/ml and then treated with different dosages of MoWa or left untreated (control). Treated *S. aureus* bacteria were incubated for 1 h. All viable counts of cultures of *S. aureus* strains incubated with MoWa at a concentration of 1/0.04 M were under the limit of detection of 10 cfu/ml (**Table 3** and **Figure 3**) and all but NRS242 were also eradicated with MoWa at 0.52/0.02 M (**Figure 3**). Incubating MRSA or MSSA strains for 1 h with 0.26/0.01 M have almost no effect in *S. aureus* viability. Therefore, a conservative MIC_90_ of MoWa at 1/0.04 M, under the challenge conditions utilized in this study, was established.

**Table 3:** Antimicrobial effect of MoWa (MIC_90_), levulinic acid or SDS.

Strain	Untreatedcfu/ml	Treatments^#^		
		MoWa* (1 M/0.04 M) cfu/ml	LA (1 M)cfu/ml	SDS (0.04 M) cfu/ml
NRS408	5.00E+05	<10	6.00E+05	5.00E+05
NRS049	3.50E+05	<10	4.00E+05	3.50E+05
NRS236	3.13E+05	<10	1.20E+05	3.88E+05
NRS230	3.30E+05	<10	<10	2.60E+05
NRS242	3.15E+05	<10	<10	2.77E+05

^#^Bacteria were treated for 1 h, and all experiments were performed in a 6-well plate format. *MIC_90_, experiments repeated two times with similar results.

Parallel to the above experiment, we also assessed whether the individual components, at a concentration utilized in MoWa, would have an antimicrobial effect against *S. aureus s*trains. When three out of five *S. aureus* strains were incubated with either LA (1 M) or SDS (0.04 M), the density did not significantly change after the one h incubation period (**Table 3**). The density of strains NRS230 and NRS242, however, was below the limit of detection (<10 cfu/ml) when incubated with LA (1 M) alone but not with SDS (0.04 M). Together these experiments demonstrated that the mixture of SDS (1 M) and levulinic acid (0.04 M) (i.e., MoWa) is more effective on eradicating MRSA and MSSA strains, that it separate components, when incubated at 37°C for 1 h, under laboratory conditions.

### Rapid Eradication of MRSA Biofilms From Contaminated Surfaces by Treatment With MoWa

**Figure 4:**
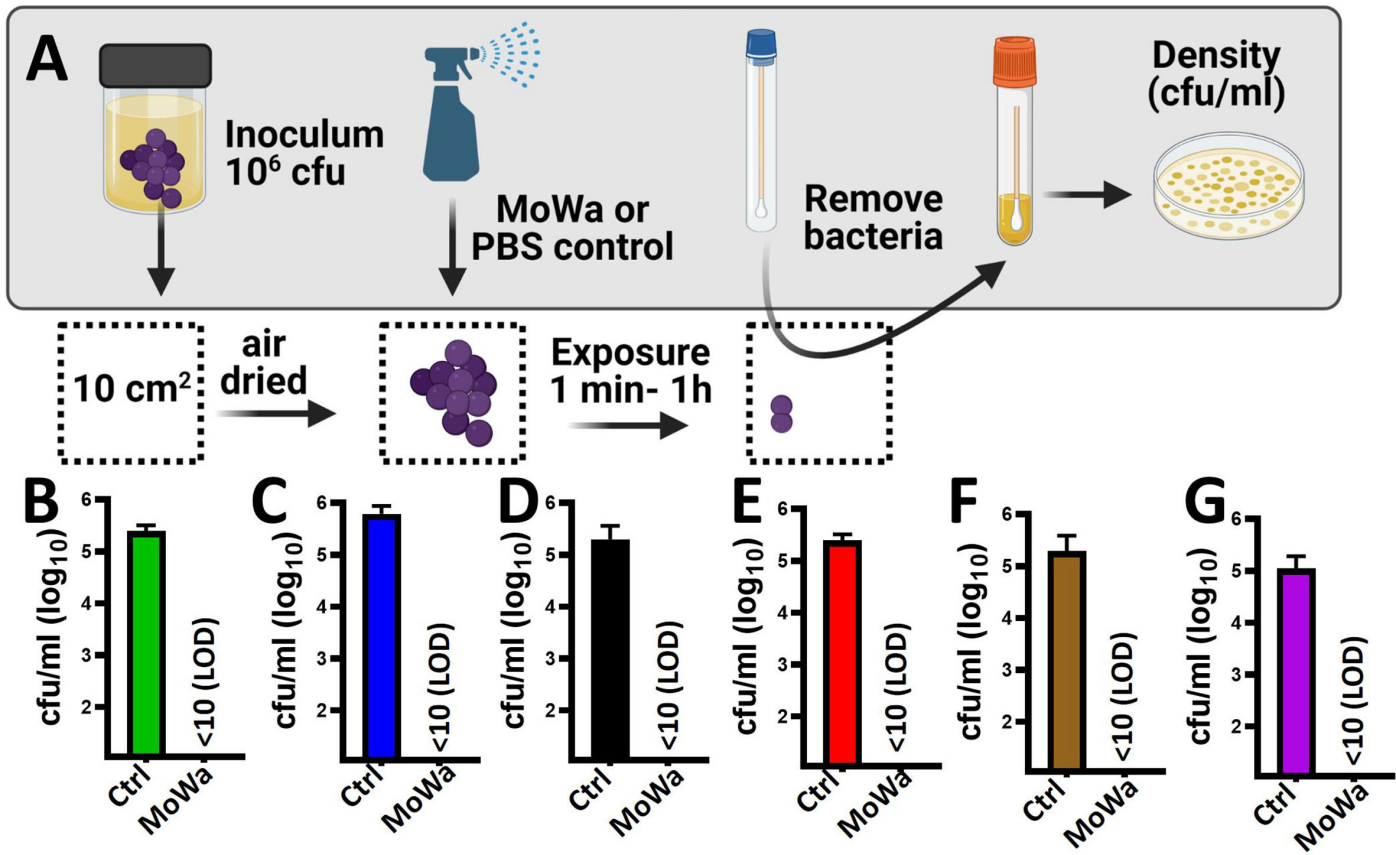
MoWa efficiently decontaminate surfaces containing MRSA and MSSA strains. **(A)** Sterilized surface of 10 cm2 was spiked with ~10^6^ cfu S. aureus strain and air-dried during 1 h. Contaminated surfaces were then sprayed with PBS (control) or with a mixture levulinic acid (LA) and SDS at a concentration of 1/0.04 M (MoWa). Bacteria were removed from MoWa treated, or mock-treated, surfaces 1 min post-treatment and immediately diluted and plated onto BHI agar plates containing 7% of NaCl to obtain colony counts (cfu/ml). Strains tested were **(B)** NRS384, **(C)** NRS408, **(D)** NRS49, **(E)** NRS236, **(F)** NRS230 or **(G)** NRS242. Error bars represent the standard errors of the means calculated using data from at least three independent experiments.

Given that MoWa is intended to be utilized as a disinfectant, we developed a model to assess the eradication of MRSA and MSSA biofilms from contaminated surfaces. To develop the model a laboratory bench made of phenolic resin was divided into areas of 10 cm^2^. The surface area was pre-disinfected with 10% bleach followed by removal of bleach with a suspension of 70% methanol and then air dried for ~1 h. A fresh suspension of an early log phase culture of MRSA strain NRS384 (USA300) was dropped and homogenized on the surface to then let the suspension dry for ~1 h (**Figure 4A**). The contaminated surfaces were sprayed with ~780 µl (median) of MoWa, 1/0.04 M, or with PBS as a control. To collect bacteria, a sterile swab was introduced into BHI broth, and the surface was swabbed as described in *Materials and Methods*. MoWa-treated surfaces were swabbed after 1 min (**Figure 4**), 5 min, 30 min, and 1 h (not shown) post treatment while mock-treated and untreated contaminated surfaces were swabbed 1 h post-inoculation. While untreated contaminated surfaces contained a density of the MRSA strain of 2.5 × 10^5^ cfu/ml (NRS384) or 5.0 × 10^5^ cfu/ml (NRS408), those treated with MoWa significantly reduced the bacterial density below the limit of detection (<10 cfu/ml) at 1, 5, 30 min, and 1 h post-exposure (**Figures 4B, C** and not shown), indicating that MoWa had eradicated the MRSA strain from the contaminated surface. A similar eradication of surfaces contaminated with other MRSA and MSSA strains was observed when MoWa was sprayed and incubated for 1 min (**Figures 4D–G**).

### Disinfectant Activity of MoWa Against Other Nosocomial Pathogens

**Figure 5:**
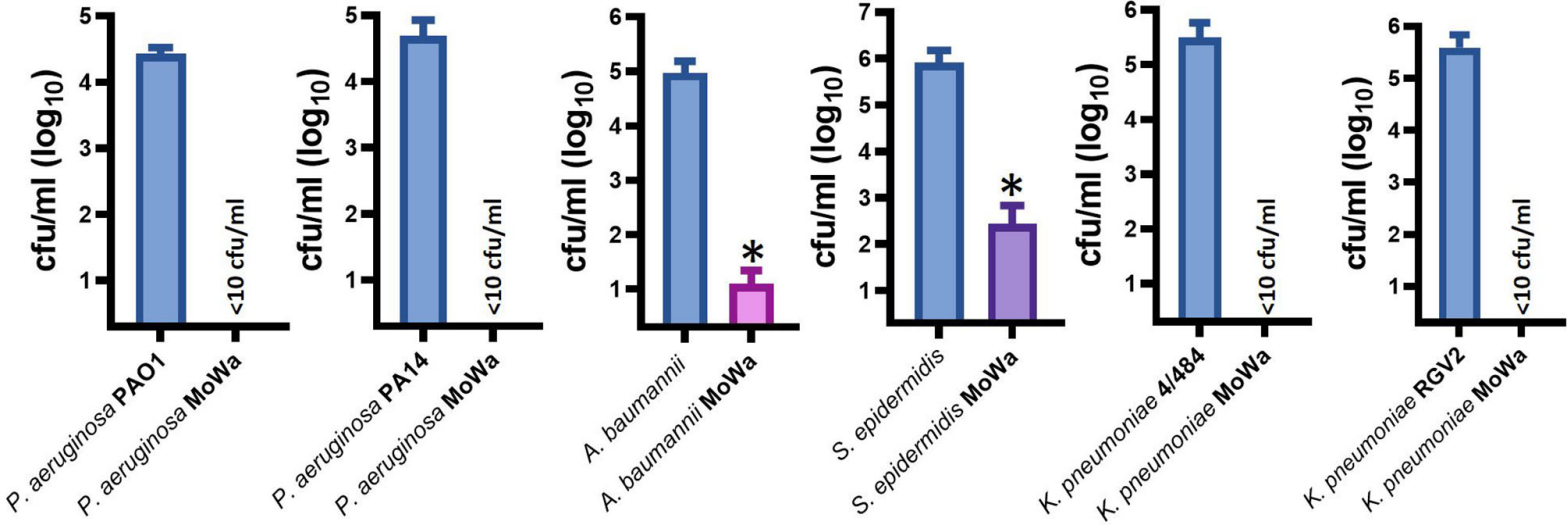
MoWa efficiently decontaminate surfaces containing nosocomial pathogens. The indicated bacterial strain was inoculated in a 10-cm2 sterile surface and let dry for ~1 h. Contaminated surfaces were sprayed with PBS (control) or with a mixture levulinic acid (LA) and SDS at a concentration of 1/0.04 M. Bacteria were removed from MoWa-treated or mock-treated surfaces after 1 min and immediately diluted and plated onto BHI agar plates to obtain colony counts (cfu/ml). Error bars represent the standard errors of the means calculated using data from at least three independent experiments. *p<0.05 compared to control.

Besides *S. aureus*, other pathogens, such as *Pseudomonas aeruginosa*, *K. pneumoniae*, *A. baumannii*, and *S. epidermidis*, are a main cause of nosocomial infection (Magill et al., 2014; Magill et al., 2018; Cruz-Lopez et al., 2020). We therefore assessed antimicrobial activity of MoWa against these nosocomial pathogens. Abiotic surfaces were contaminated with individual cultures of these pathogens and treated with the MIC_90_ of MoWa (1/0.04 M) that eradicated MRSA and MSSA strains. We challenged two reference strains of *P. aeruginosa*, PA01 and PA14; whereas the density of the untreated control was >2.5 × 10^4^ cfu/ml, the density of cultures of those abiotic surfaces contaminated with *P. aeruginosa* strains and treated with MoWa was below the limit of detection of <10 cfu/ml, indicating that bacteria had been completely eradicated (**Figure 5**). Similarly, the density of surfaces contaminated with *A. baumannii* ATCC strain 17978 was reduced ~99.99%, when comparing the mean density of the untreated control (9.32 × 10^4^ cfu/ml) against MoWa-treated contaminated surfaces (2.0 × 10^1^ cfu/ml). We also assessed MoWa antimicrobial effect against the most prevalent coagulase-negative staphylococci, *S. epidermidis*. The reduction of the density 1-min post treatment (mean, 2.72 × 10^2^ cfu/ml) reached ~99.92% compared to the untreated control (mean, 8.08 × 10^5^ cfu/ml). Finally, we challenged two *K. pneumoniae* strains isolated from nosocomial infections in our previous study (Ostria-Hernandez et al., 2018), one of them strain 4/484 is a multidrug-resistant bacterium (Ostria-Hernandez et al., 2018). MoWa completely eradicated surfaces contaminated with these two strains (**Figure 5**).

## Discussion

We have thoroughly demonstrated in this study that a mixture of LA and SDS rapidly killed *S. aureus*, including MRSA and MSSA strains, and some of the most important nosocomial pathogens throughout the world. There was a clear dose-response, time dependence, killing kinetics of *S. aureus* strains by MoWa. For example, planktonic cells were eradicated within 24 h with a very low concentration (8.2/0.3 mM), whereas biofilms grown at a higher density were killed in 24 h with (32/1.3 mM), and it required of 1 M/0.04 M to kill bacteria within 1 h; this latter was established as the MIC_90_. This MIC_90_ not only killed several MRSA and MSSA strains using the *in vitro* assay with BHI broth but also eradicated MRSA and MSSA strains from contaminated surfaces after 1 min of exposure.

While not assessed in the current study, the concentration of LA (1 M) or SDS (0.04 M) that reached a MIC_90_ should be safe for individuals that would potentially utilize MoWa to decontaminate hospital surfaces, and others that might be present in the hospital facility. The safety of LA and SDS for humans has been widely assessed, and therefore, those two compounds were designated by the US Food and Drug Administration (FDA) as GRAS given they can be directly added to food as a flavoring substance (LA), or as a multipurpose additive (SDS) (Zhou et al., 2019; Administration FFaD, 2021). Furthermore, the investigated LD_50_ of SDS is 977 mg/kg, thereby it will require 233 M of SDS to affect an individual weighing 70 kg. On the other hand, the LD_50_ of LA is 1850 mg/kg (Anonymous, 1979), which is the equivalent to 15 M and therefore it will require 1050 M of LA to affect an average individual. Therefore, the contact-killing MIC_90_ of 1/0.04 M of MoWa is ~1,000-fold, or ~5,825-fold, lower than that concentration required to reach a LD_50_ in animal models. At the concentration used in MoWa, levulinic acid has a light fruity fragrance, it is in fact a common additive to foods (Administration FFaD, 2021) and therefore its smell will not represent a negative factor to discourage its use. Neither sensorial (i.e., smell, color, or other sensory aspects) nor safety concerns were identified when a similar solution of LA and SDS was utilized to decontaminate strawberries (Zhou et al., 2017). A similar MoWa formulation did not cause adverse effects in mice after a 2-week period of treatment by the oral route (Wang et al., 2012).

A very interesting observation in the current study that deserves further investigation were those experiments demonstrating that, at the concentration mixed in MoWa, the individual compounds were not able to kill MRSA and MSSA strains. Levulinic acid (C_5_H_8_O_3_) is a 4-oxopentanoic acid containing two functional groups, carboxylic acid and ketones. These two groups certainly provide pathways to further reactions. SDS is a detergent used to solubilize biological membranes by forming micelles with membrane phospholipids; this occurs at a critical micelle concentration (CMC) of SDS of 0.3 mM per mole of phospholipids (Tan et al., 2002). The presence of a five-carbon molecule, i.e., levulinic acid, might have decreased the CMC, thus increasing the solubilization of the bacterial membrane at a lower saturating concentration of SDS. An additional hypothesis might be that once SDS have permeabilized the bacterial membrane(s), LA could trigger downstream signaling events at the intracellular level and leading to the observed rapid death of bacteria.

The infectious dose of *S. aureus* strains that causes disease varies but can be as low as 10 bacteria to several thousand cfu (Dancer, 2008). In our life-like contamination model, we infected bacteria spanning most, if not all, bacterial densities observed in studies assessing the contamination of surfaces in hospital environments. MoWa successfully eradicated all surfaces that had been contaminated with ~1 × 10^6^ cfu, within 1 min of exposure, thereby it is expected that this disinfectant eradicates contamination with a lower density. It has been reported that *S. aureus* strains can be carried in the nose by ~25% of hospitalized patients of which <1% corresponds to MRSA strains (Weterings et al., 2019). In China, ~20% of healthcare workers carry MRSA strains while the prevalence in the general population was ~28% (Wu et al., 2019). It is therefore expected that 30% of individuals in a hospital, whether patients, their family member, or hospital staff carry *S. aureus*. Transmission of these strains from nose to hands occurred in 100% of those carrying staphylococci in the upper airways and serves as a persisting vector of contamination (Kramer et al., 2006). Our initial characterization demonstrated antibacterial activity against 100-fold more cfu than that required to initiate an infectious process and eradication of bacteria contaminating a surface at a density of ~10^4^ cfu/cm^2^ as rapid as 1 min post-treatment with MoWa.

Levulinic acid plus SDS-based intervention approaches for reducing contamination with shiga toxin-producing *E. coli* (STEC) have also proven effective in reducing the bacterial density, although in those studies, the temperature has been a factor for the reduction of bacterial viability with the greatest reduction obtained at temperatures >8°C (Ortega et al., 2011; Zhou et al., 2019). This was not a factor in our study as our *in vitro* test was conducted at 35°C, and the ambient temperature, when assessed using the surface-contaminated model, was ~20°C. The temperature should not represent a factor whether MoWa is utilized in hospital environments where the ambient temperature should be around 20°C but certainly not below 10°C. A similar formulation of LA and SDS has been used overall to disinfect bacteria contaminating pecans (Beuchat et al., 2012; Beuchat et al., 2013), cantaloupes (Webb et al., 2013), or beef cheek meat (Schmidt et al., 2014) and from food slicers (Chen et al., 2014). Besides environmental contamination, LA/SDS removed biofilms made by oral streptococci (Wang et al., 2012). Given the potential safety of MoWa, and its proven antimicrobial effect against MRSA strains, dermatological use of MoWa for the prophylactic use, or the treatment of, MRSA-induced skin infection is envisioned. To the best of our knowledge, until the current study, this LA/SDS solution has not been assessed to disinfect clinical settings contaminated with bacterial nosocomial pathogens. It will be important also to assess in the future whether MoWa can inactivate SARS-CoV-2 viruses, since it has demonstrated activity against the influenza A virus and surrogated human noroviruses (Cannon et al., 2012; Aydin et al., 2013).

Antimicrobial activity against MRSA and *E. coli* has also been demonstrated, with a similar efficacy to that presented in the current study, for antimicrobial formulations containing chlorhexidine gluconate and/or isopropyl alcohol (Campos et al., 2012; Hendry et al., 2012). Ethanol wipes, and a portable UV radiation device, were also effective to eradicate contamination by MRSA and other nosocomial pathogens whereas spraying a solution of 70% ethanol only reduced the density of MRSA (Panousi et al., 2009; Umezawa et al., 2012). The advantage of MoWa against other available sanitizing for hospital environments includes the known safety of its components (Administration FFaD, 2021), the availability of LA and SDS during shortages of disinfectants, such as the one we are experiencing in the current Covid-19 pandemic (Berardi et al., 2020), and the lower price for eradicating nosocomial pathogen from contaminated surfaces. Compared to alcohol-based disinfectants, treating a surface by spraying of MoWa (~780 µl) will cost >10-fold cheaper than a similar treatment of the contaminated surface with an alcohol-based solution or alcohol wipes (i.e., MoWa US $0.010 cents compared to a solution of 70% ethanol US $0.100 cents or alcohol wipes US $0.300 cents each wipe).

In summary, MoWa showed a high efficacy to decontaminate abiotic surfaces infected with some of the most important nosocomial pathogens such as MRSA strains, MSSA strains, *P. aeruginosa*, *K. pneumoniae*, *A. baumannii*, and *S. epidermidis*. This new disinfectant for hospital environments bear such a great potential to reduce the burden of morbidity and mortality in hospitals given the shortage of disinfectants and because of the availability and low price of its components.

## Data Availability Statement

The raw data supporting the conclusions of this article will be made available by the authors, without undue reservation.

## Author Contributions

All authors listed have made a substantial, direct, and intellectual contribution to the work and approved it for publication.

## Conflict of Interest

The authors declare that the research was conducted in the absence of any commercial or financial relationships that could be construed as a potential conflict of interest.
